# Silencing Early Viral Replication in Macrophages and Dendritic Cells Effectively Suppresses Flavivirus Encephalitis

**DOI:** 10.1371/journal.pone.0017889

**Published:** 2011-03-15

**Authors:** Chunting Ye, Sojan Abraham, Haoquan Wu, Premlata Shankar, N. Manjunath

**Affiliations:** Paul L. Foster School of Medicine, Center of Excellence in Infectious Diseases, Texas Tech University Health Sciences Center, El Paso, Texas, United States of America; Naval Research Laboratory, United States of America

## Abstract

West Nile (WN) and St. Louis encephalitis (SLE) viruses can cause fatal neurological infection and currently there is neither a specific treatment nor an approved vaccine for these infections. In our earlier studies, we have reported that siRNAs can be developed as broad-spectrum antivirals for the treatment of infection caused by related viruses and that a small peptide called RVG-9R can deliver siRNA to neuronal cells as well as macrophages. To increase the repertoire of broad-spectrum antiflaviviral siRNAs, we screened 25 siRNAs targeting conserved regions in the viral genome. Five siRNAs were found to inhibit both WNV and SLE replication in vitro reflecting broad-spectrum antiviral activity and one of these was also validated in vivo. In addition, we also show that RVG-9R delivers siRNA to macrophages and dendritic cells, resulting in effective suppression of virus replication. Mice were challenged intraperitoneally (i.p.) with West Nile virus (WNV) and treated i.v. with siRNA/peptide complex. The peritoneal macrophages isolated on day 3 post infection were isolated and transferred to new hosts. Mice receiving macrophages from the anti-viral siRNA treated mice failed to develop any disease while the control mice transferred with irrelevant siRNA treated mice all died of encephalitis. These studies suggest that early suppression of viral replication in macrophages and dendritic cells by RVG-9R-mediated siRNA delivery is key to preventing the development of a fatal neurological disease.

## Introduction

West Nile (WN), Japanese B encephalitis (JE) and St. Louis encephalitis (SLE) viruses are mosquito-borne flaviviruses that can cause a devastating acute neurological illness with up to 30% mortality and permanent neurological disabilities in the survivors [1]. There has been a steady increase in the number of WNV infections in the US since it first appeared in 1999 [2], [3], [4]. WNV has been classified as potential category B bioterrorism agent by NIAID and there is no effective treatment for this infection. The recently emerging RNA interference (RNAi) technology appears to have a great potential in antiviral therapeutics (reviewed in [5], [6], [7]. However, currently the major limitation for therapeutic use of siRNA is the short serum half-life and poor cellular uptake of siRNA [8]. Thus, delivering effective quantities of siRNAs into the right target cells in vivo through clinically feasible methods represents a major challenge for the successful development of RNAi-based therapeutics [9]. Our recent studies suggest that a small peptide derived from the rabies virus glycoprotein fused to a highly positively charged 9-mer polyarginine residues (RVG-9R) can provide a tool for siRNA delivery to neuronal cells as well as macrophages [10], [11]. Intravenous injection with RVG-9R-complexed siRNA in mice could reduce the LPS induced TNF-α production by macrophages in blood as well as by microglia in the brain, leading to a significant reduction in neuronal apoptosis [11]. Since flaviviruses are thought to first multiply in dendritic cells and macrophages before spreading to the brain [12], [13], [14] in this study we tested if RVG-9R is able to deliver siRNA to macrophages to suppress the early virus replication in these cells. In addition, to expand the repertoire of broad-spectrum siRNAs capable of suppressing multiple flaviviruses, we also tested a panel of 25 siRNAs targeting relatively conserved genomic regions.

## Results

### Identification of broad-spectrum antiflaviviral siRNAs

**Figure 1:**
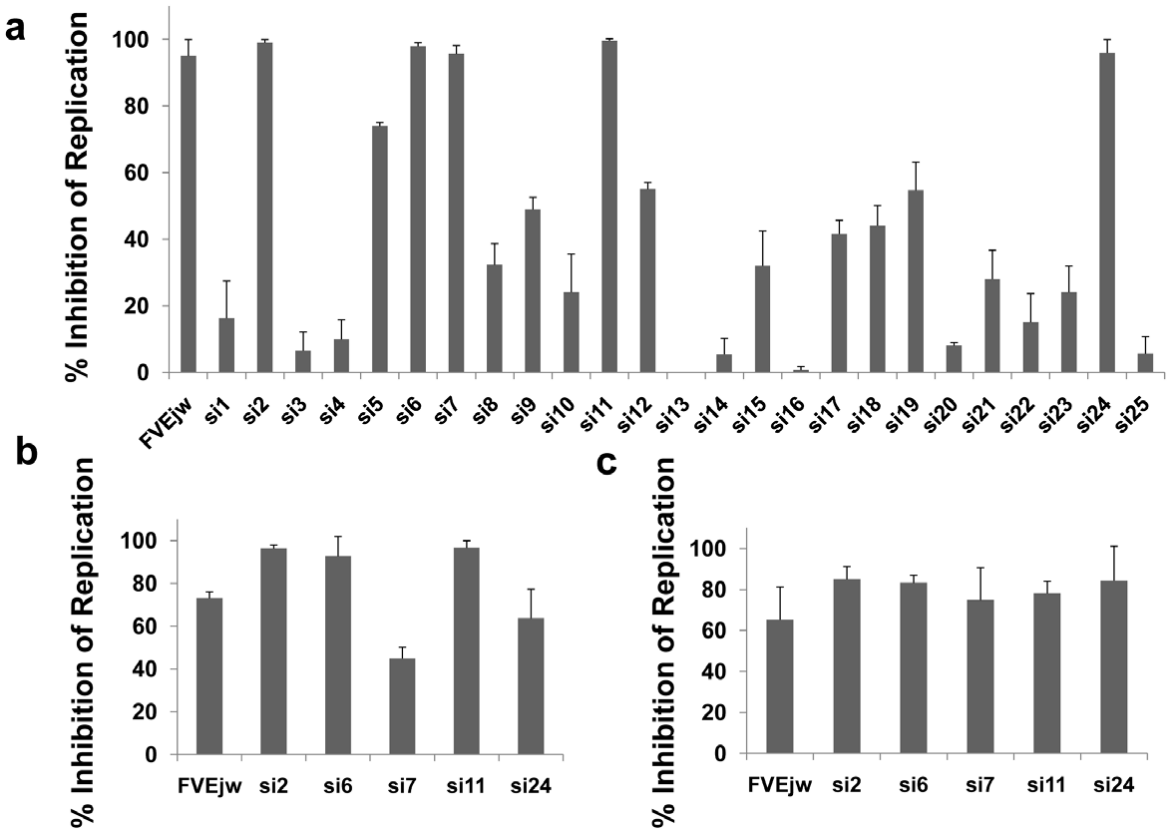
Protection against WN and SLE viral infection by various siRNAs in BHK21 cells. a) BHK-21 cells were transfected with indicated siRNAs using lipofectamine 2000, infected with WNV (1moi) 24 h later and tested for virus replication 72 h after infection by FACS analysis. b) Cells were first infected with WNV, transfected with indicated siRNAs 24 h later and tested for virus replication as in a). c) Indicated siRNAs were tested for inhibition of SLE replication as in a). All experiments were done in triplicate in 2 independent experiments and the error bars indicate SD.

We have previously shown that a siRNA (FVE^jw^) targeting highly conserved region in the viral envelope gene could inhibit the replication of both JEV and WNV [15]. However, it has been suggested that instead of a single siRNA, using a combination of 2–3 siRNAs targeting multiple genomic regions may be ideal to enhance efficacy and reduce viral escape as well as off-target effects [16]. Thus, to identify additional siRNAs with broad-spectrum antiviral activity, we tested 25 siRNAs targeting highly conserved regions (Table 1) in the flaviviral genome. We first tested all 25 siRNAs for inhibition of WN infection using FVE^jw^ siRNA described earlier as positive control. BHK-21 cells were transfected with individual siRNAs using lipofectamine reagent 24 h before challenge with WNV and tested for virus replication 72 h after challenge by FACS analysis using anti-West Nile Virus/Kunjin envelope specific monoclonal antibody (clone 3.67G, Chemicon) as described earlier [15]. Out of these 25 siRNAs, 5 could inhibit virus replication by more than 80% (Fig. 1a). To test if these siRNAs are also effective when introduced postinfection, BHK-21 cells were first infected with WNV followed 24 h later with siRNA transfection and analyzed for virus replication 72 h later. As shown in Fig. 1b, all 5 siRNAs inhibited viral replication to varying degrees with the best efficacy of 80% inhibition seen with si2, si6 and si11 siRNAs. The 5 effective siRNAs were also tested for broad-spectrum antiviral activity. Since the target regions are also conserved in the SLE, we tested the siRNAs for protection against SLE infection. As shown in Fig. 1c, 60–80% inhibition of SLE virus replication was seen with these siRNAs. Thus, the 5 siRNAs constitute siRNAs with broad-spectrum antiviral activity effective across at least two viral species.

**Table 1:** Details of siRNAs targeting conserved regions of West Nile virus.

siRNA designation	Guide strand sequence	Region in WNV genome	Nt position
Si-01	ucgcauuccguuguguuuudTdT	Env	2980–2996
Si-02	accgcguuuuagcauauugdTdT	Core	138–156
Si-03	ccgcguuuuagcauauugadTdT	Core	137–155
Si-04	ccuagcauccauccaaucgdTdT	Pre M	886–902
Si-05	gcguuuuagcauauugacadTdT	Core	135–153
Si-06	ugacucuccaaugucacagdTdT	NS5	8103–8121
Si-07	caguugaagcuguaugccgdTdT	Pre M	958–974
Si-08	aaugcuccccuuuccaaacdTdT	Env	1287–1305
Si-09	cugugugauccaggacauudTdT	NS1	2323–2340
Si-10	augcuccccuuuccaaacadTdT	Env	1286–1304
Si-11	cgcguuuuagcauauugacdTdT	Core	136–154
Si-12	cguuuuagcauauugacaadTdT	Core	135–152
Si-13	augugucaaugcuccccuudTdT	Env	1294–1312
Si-14	gugaagguguucagggcaudTdT	NS5	9502–9518
Si-15	ucaaugcuccccuuuccaadTdT	Core	76–94
Si-16	caaugcuccccuuuccaaadTdT	Env	1288–1306
Si-17	gugucaaugcuccccuuucdTdT	Env	1292–1310
Si-18	ucccugugugauccaggacdTdT	Env	2325–2343
Si-19	ugugucaaugcuccccuuudTdT	Env	1293–1311
Si-20	ugugugauccaggacauucdTdT	Env	2323–2339
Si-21	gacucuccaaugucacagadTdT	NS5	8102–8120
Si-22	guuuuagcauauugacaacdTdT	Core	135–151
Si-23	cucuccaaugucacagagcdTdT	NS5	8100–8118
Si-24	ucuccaaugucacagagcadTdT	NS5	8099–8117
Si-25	acccaguacaucucaugugdTdT	NS5	8320–8336

### In vivo efficacy of siRNAs

**Figure 2:**
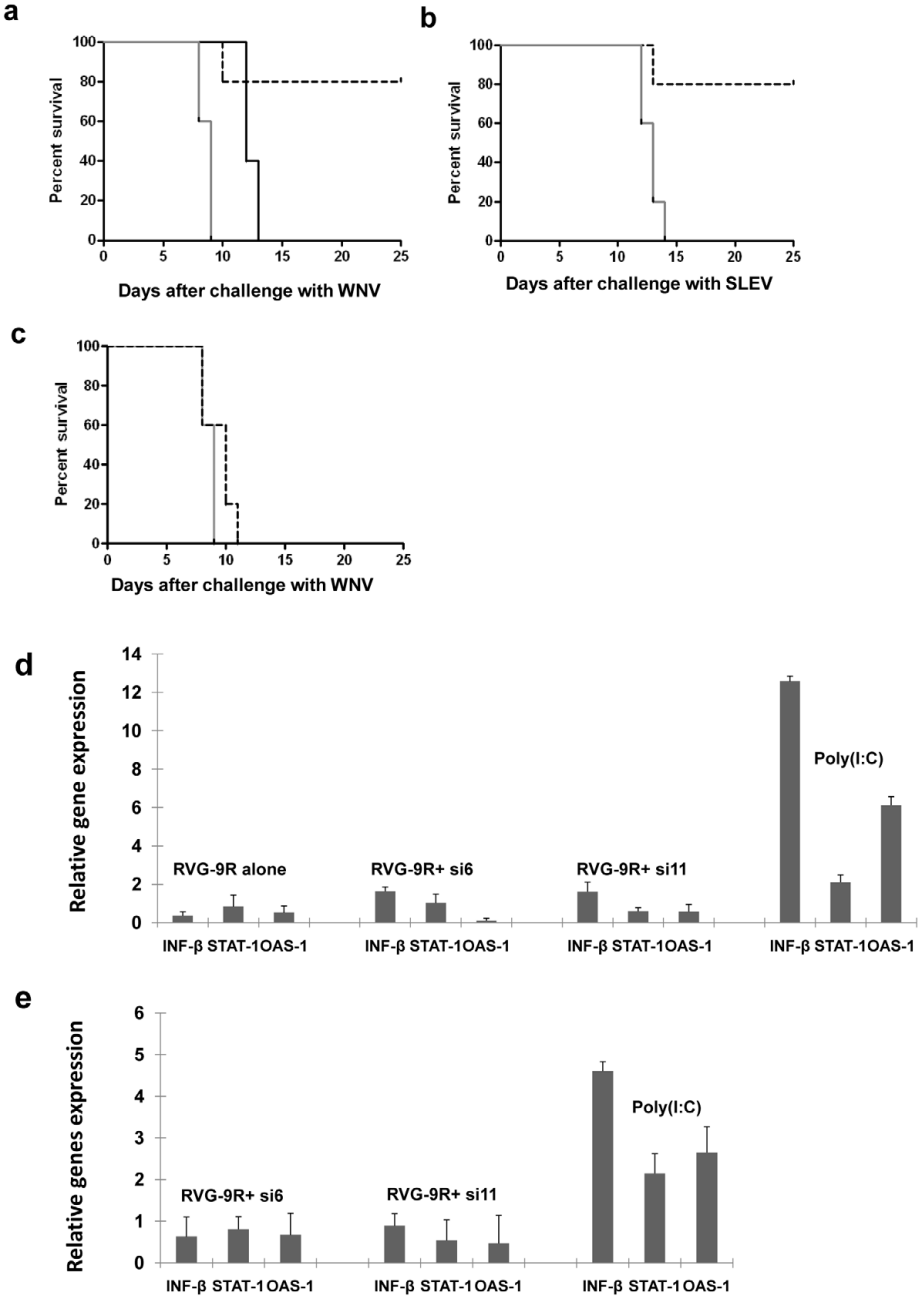
Intravenous treatment with antiviral siRNA/RVG-9R complex before infection protects mice against WNV and SLEV induced encephalitis without inducing an interferon response. Mice were treated intravenously with either control siLuc, or si6 or si11, complexed with RVG-9R 15 h before infection with 5 LD_50_ of WNV (a) or SLEV (b), the siRNA treatment repeated 4 and 24 h after infection and monitored for survival over time. Black solid line represents si11; black broken line, si6; grey solid line, siluc. n = 10/group. c) Mice were treated with the control siLuc (solid black line) or si6 siRNA (broken line) daily from 2–5 days after WN infection and monitored for survival. d) Raw 264.7 cells were treated with indicated siRNAs complexed with RVG-9R or as positive control, with poly (I:C) and 4 h later, the cellular RNA was tested for upregulation of IFN β, OAS-1, and STAT-1 mRNAs by qRT-PCR (n = 3). e) Mice were iv injected with poly (I:C) or siRNAs complexed with RVG-9R and 1 h later, their total RNA from spleen cells were tested by qRT-PCR (3 mice/group).

We selected two siRNAs, si6 and si11 for testing their ability to suppress WN and SLE infection in vivo. Si6 targets the WNV NS5 gene, whereas Si11 targets the WNV core protein C. We have previously reported that intravenous delivery of siRNA complexed with RVG-9R can protect mice from JEV-induced encephalitis. Thus we used the same system to test effectiveness against WN infection. Unlike wild type mice, immunodeficient mice including B-cell-deficient mice are uniformly susceptible to peripheral infection with flaviviruses [17]. Thus, after confirming that intraperitoneal infection with our B956 strain of WNV induces 100% mortality in B cell-deficient mice, we tested the treatment with si6 and si11. We infected groups of B cell-deficient mice (10 mice/group) with WNV intraperitoneally and treated them with iv injection of RVG-9R/siRNA complex. The test and the control irrelevant luciferase siRNAs (50 µg/dose/mouse) were administered 15 hours before infection, the siRNA treatment repeated 6 and 24 hours after infection (a total of 3 injections) and the mice were observed for survival for 25 days. All mice treated with the control siRNA/RVG-9R developed a typical neurologic disease and died within 9 days showing that the irrelevant siRNA or RVG-9R has no influence on the course of the disease. In sharp contrast, 80% of mice treated with si6/RVG-9R were protected, with mice living without sickness during the entire course of observation for 25 days (Fig. 2a). The mice treated with si11/RVG-9R showed only partial protection in that they showed a delay in the development of disease but all mice died by 13 days. Next we tested si6 for protection against SLE infection, with infection and siRNA treatment done as described earlier. Similar to the results seen with WNV infection, si6 siRNA treatment showed nearly 80% protection (Fig. 2b). Thus, si6 siRNA appears to have broad-spectrum antiviral activity in vitro and in vivo. Although siRNA treatment started before infection was protective, when the treatment was initiated 2 days after infection, it failed to afford any protection (Fig. 2c), suggesting that suppression of early viral replication is important to prevent the neuronal disease. We also confirmed that si6 and si11 siRNAs do not induce interferon response both in vitro and in vivo. For this, Raw 264.7 cells were treated with siRNAs or as positive control, poly (I:C) and 4 h later, the cellular RNA was tested for upregulation of IFN β, OAS-1, and STAT-1 mRNAs by qRT-PCR. Although all these mRNAs were elevated in poly (I:C) treated cells, cells treated with siRNAs did not show an elevated expression of IFN or interferon inducible genes (Fig. 2d). To confirm this in vivo, C57BL/6J mice were treated with poly (I:C) or siRNAs complexed with RVG-9R and 1 h later, their spleen cells were tested for IFN response as described above. Even in this case, while poly (I:C) elicited IFN response, siRNAs did not show an elevation of IFN or related genes (Fig. 2e). Taken together, our results suggest that si6 and si11 siRNAs suppress viral replication by RNAi and not due to an IFN response.

### siRNA delivery to macrophages and dendritic cells is critical to suppress WNV encephalitis

**Figure 3:**
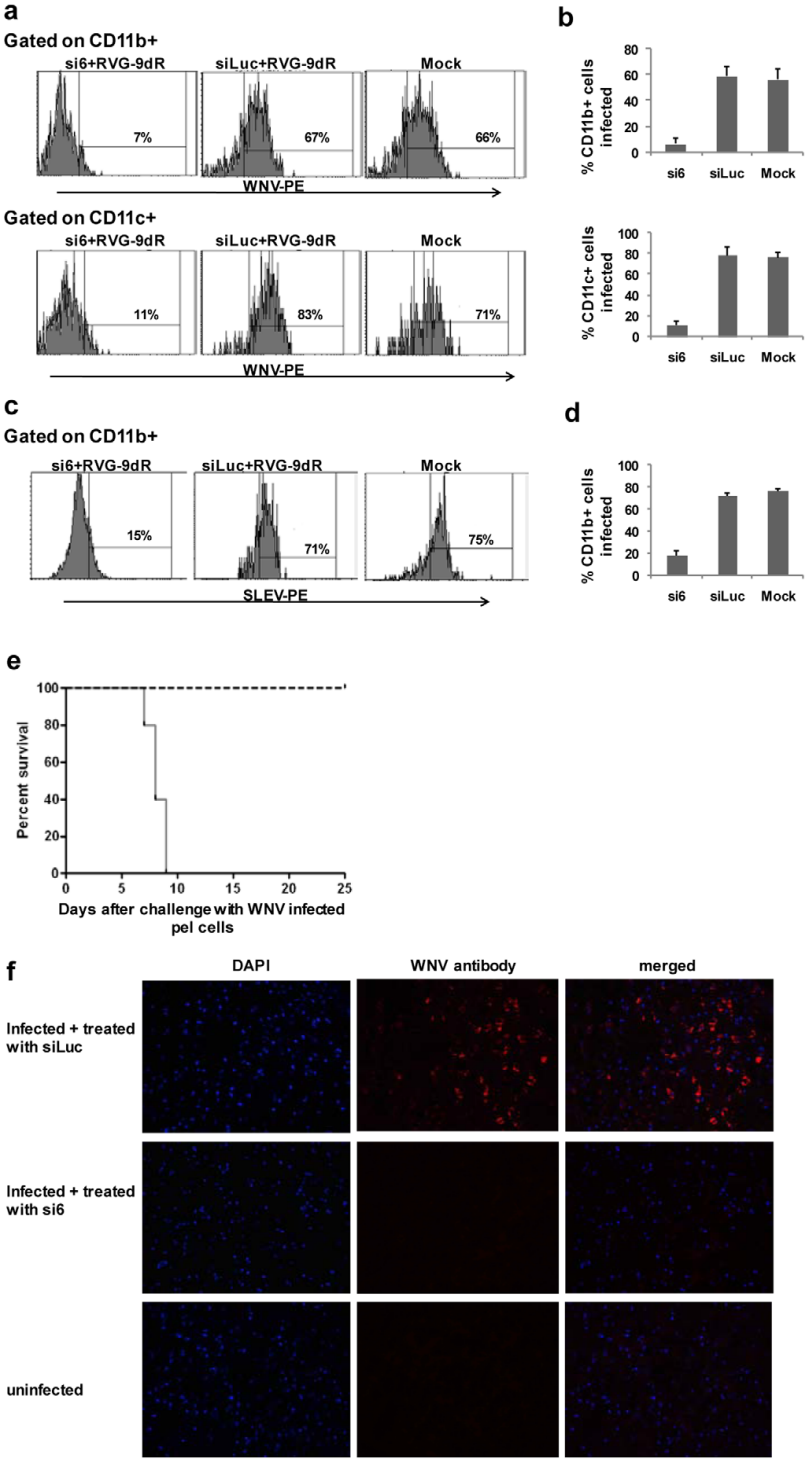
RVG-9dR delivered siRNA suppresses early WNV replication in macrophages and dendritic cells. Mice were infected with WNV and either not treated (mock) or treated with the control siLuc or si6 siRNA as in Fig. 2a and 3 days after infection, the peritoneal exudate cells tested for virus replication in CD11b and CD11c gated cells by flow cytometry. A representative histogram (a) and cumulative data from 3 mice (b) are shown. Error bars represent SD. c,d) Mice were infected with SLE, treated with control or si6 siRNA and their peritoneal exudate cells examined for virus infection 3 days after infection as in b. A representative histogram (c) and cumulative data from 3 mice (d) are shown. Error bars represent SD. e) Immunomagnetically isolated CD11b+ macrophages from mice in Fig. 2a were transferred to new mice ip and the mice followed for survival over time. Solid black line represents si6 treated mice and broken line indicates siLuc treated mice. f) Photomicrographs of brain sections from mice in Fig. 3c stained with WNV envelope-specific antibody and DAPI (magnification = 20X).

During WNV infection, the virus is thought to initially replicate in dendritic cells and macrophages before being seeded into the brain between day 3–4 after infection and then, rapidly multiples in the brain cells leading to fulminant encephalitis and death by day 8–9. We have reported that RVG-9R mediates siRNA delivery to neuronal cells as well as to macrophages and dendritic cells [11]. Thus, we tested if siRNA delivery to macrophages/DC is enough to suppress early virus replication and thereby prevent neuronal disease. For testing this, we infected mice with WNV ip and treated iv with si6 or the control siLuc complexed RVG-9R as describe above. Peritoneal exudate cells harvested on day 3 were analyzed for infection by FACS analysis after staining with CD11b, CD11c and anti-West Nile Virus/Kunjin envelope antibody. As shown in Fig. 3a and b, a majority of CD11b+ macrophages and CD11c+ DC from control mice were infected, while macrophages and DC from si6 treated mice were largely protected. We also confirmed protection in peritoneal exudate macrophages by si6 treatment following SLE infection (Fig. 3c,d). Next, we immunomagnetically isolated CD11b+ cells from the peritoneal exudate cells obtained from control and si6 siRNA treated mice and transferred them to new B cell-deficient hosts to detect transmission. All mice transferred with macrophages from control siRNA treated mice developed a neurological disease and died by day 9 while those transferred with macrophages from si6 treated mice all survived with no illness (Fig. 3e). Moreover, brain sections stained with WNV envelop antibody revealed absence of infected cells in mice transferred with CD11b cells from si6 treated mice, compared to that from the control siLuc treated mice (Fig. 3f). Thus, RVG-9R-mediated siRNA delivery to macrophages/DC is sufficient to prevent the initial viral replication in these cells and thereby prevent viral dissemination to the brain.

## Discussion

In this study, we have identified several siRNAs with broad-spectrum antiflaviviral activity and shown that suppression of early viral amplification in macrophages/dendritic cells is sufficient to prevent fatal encephalitis.

Several previous studies have shown that siRNAs can effectively inhibit the replication of several flaviviruses including dengue, West Nile and Japanese encephalitis viruses in vitro (reviewed in [6]. One previous study also showed effectiveness in vivo in a mouse model for West Nile virus using a nonphysiological hydrodymanic injection [18]. However for actual clinical treatment, effective and nontoxic methods must be developed to deliver siRNA to susceptible cells in vivo. Moreover, since clinical symptoms often overlap and laboratory diagnosis takes time, it would be better to design siRNAs with broad-spectrum activity that can suppress related viruses across species. We have previously shown that a single siRNA targeting a highly conserved region in the domain 2 of the envelope protein can suppress infection with both WN and JE viruses [15]. However, multiple siRNAs with broad spectrum activity will be desirable for a number of reasons: a combination of siRNAs targeting multiple regions reduces the concentration of individual siRNAs and thereby reduces toxicities induced by off-target effects [16]; multiple siRNAs also reduce the chances of viral escape [19], [20]; although many regions in flaviviruses are highly conserved at the amino acid level, they can differ at nucleotide level and since small sequence changes can affect RNAi, using multiple siRNAs also help extend the broad-spectrum activity by covering viral strains showing small nucleotide differences. We propose that a combination of FVE^JW^ siRNA described earlier together with si6 and si11 identified in this study would serve to suppress a panel of encephalitogenic flaviviruses including WN, JE and SLV. All these siRNAs have been shown to not induce an interferon response and thus, makes an RNAi therapy possible.

One major hurdle for in vivo therapy is the lack of physiological methods for siRNA delivery to appropriate cell types in vivo. Since encephalogenic flaviviruses such as WN, JE and SLE viruses initially expand in dendritic cells and macrophages and then extensively replicate in the brain, ideally siRNA needs to be delivered to both these cell types. To this end, we have previously reported that a short peptide derived from the Rabies virus glycoprotein can bind AchR expressing neuronal cells as well as macrophages and when fused to 9R peptide, the chimeric RVG-9R peptide can bind siRNA (by charge interaction) and deliver it to these cell types. Indeed, iv treatment with FvE^jw^ siRNA complexed with RVG-9R was able to protect mice from fatal JEV-induced encephalitis. However, since RVG-9R delivers siRNA to both macrophages and neuronal cells, it was unclear as to what role silencing in macrophages played in preventing encephalitis. Our results in the present study showing that control macrophages isolated from WN infected mice initiated a fatal disease in new hosts while siRNA treated macrophages were not, clearly shows that initial suppression of viral replication in macrophages is enough to prevent viral transmission to the brain, although silencing in the brain cells might confer an added benefit to suppress any leak through virus that might enter the brain. The fact that siRNA treatment failed to protect when initiated 2 days after infection (Fig. 2c) also point to the importance of preventing initial viral replication in macrophages/dendritic cells, although inaccessibility of the viral genome for RNAi machinery because of sequestration in membranous structures might also contribute to the failure for protection beyond 2 days after infection.

Although our results suggest that inhibiting viral replication in macrophages is enough to prevent development of a neurological disease, the exact mechanism how this happens is not clear. It is possible that in the treated animals, the viral burden does not reach a level required to cross the blood-brain barrier. However, a recent study suggests that neutrophils and not macrophages constitute the major reservoir for the early viral replication [21]. Since neutrophils are also known to express AchR [22] it is possible that RVG-9R is also capable of delivering siRNA to neutrophils to suppress viral replication, although this needs to be formally tested. On the other hand, macrophages are also thought to act as “Trojan horses” to carry virus into the brain [23]. Thus, RVG-9R mediated siRNA delivery might both lead to a reduced circulating viral load as well as prevent infected macrophage seeding into the brain to suppress the development of a neurologic disease.

In summary, we have identified individual siRNAs that can suppress multiple encephalitogenic flavivirus species and shown that silencing viral replication in DC/macrophages is critical to preventing the development of neurological disease.

## Materials and Methods

### Peptides and siRNAs

RVG-9R (YTIWMPENPRPGTPCDIFTNSRGKRASNGGGGRRRRRRRRR) peptide was synthesized and purified by high-performance liquid chromatography at the Tufts University Core Facility (Boston, MA). In RVG-9R, the carboxy-terminal nine arginine residues were d-arginines. siRNAs shown in Table 1 for the initial screeing in in vitro studies were synthesized at Alnylam Pharmaceuticals. Large scale antiviral siRNAs for animal experiments including si6 and si11 as well as the siRNA targeting firefly luciferase (siLuc; 5′-CUUACGCUGAGUACUUCGAdTdT-3′) were synthesized at Dharmacon (Lafayette, CO).

### siRNA transfection and WNV suppression in vitro

Baby hamster kidney cell line (BHK21) was obtained from ATCC (Manassas, Virginia, United States) and maintained in DMEM with 10% FCS. The B956 strain of WNV was obtained from ATCC, grown, and plaque titrated using BHK21 cells. BHK21 cells were seeded in 12-well plates at 5×10^4^ per well for 12–16 h before transfection. Lipid-siRNA complexes were prepared by incubating 150 or 300 pmol of siRNA with Lipofectamine 2000 (Invitrogen, Carlsbad, California, United States) according to the manufacturer's instruction. Lipid-siRNA complexes were added to the wells in a final volume of 0.5 ml of serum-free DMEM. After incubation for 6 h, cells were washed, and reincubated in DMEM containing 10% FCS, and infected with WNV 24 h post-transfection or 24 h pre-transfection. The infection levels were monitored after 72 h by flow cytometry using anti-West Nile Virus/Kunjin envelope specific monoclonal antibody (clone 3.67G, Chemicon International, from Millipore, USA).

### Animal experiments for testing siRNA to suppress WNV and SLE

B cell-deficient mice were purchased from the Jackson Laboratories and used at 6–8 weeks of age. All mouse experiments had been approved by the TTUHSC IACUC, and animal infection experiments were performed in a biosafety level 3 animal facility at the TTUHSC. To test protection against WNV, peptide/siRNA complexes (at a peptide to siRNA molar ratio of 10∶1) were prepared in 100–200 µl of 5% glucose and injected intravenously at 50 µg of siRNA per mouse per injection 15 hours before i.p. infection with WNV or SLE (5LD_50_/mouse). The IV siRNA treatment was repeated 6 and 24 h following infection (a total of 3 injections) and the mice were observed for survival for 25 days.

### RVG-9dR delivery siRNA to macrophage and dendritic cells to suppress WNV infection in vivo

B cell-deficient mice were challenged with WNV and treated with si6 or siLuc complexed RVG-9dR as describe above. Spleen cells were isolated 3 days after challenged, stained with CD11b or CD11c antibody (BD Biosciences, USA) and anti-West Nile Virus/Kunjin envelope specific monoclonal antibody and tested by flow cytometry. Peritoneal CD11b+ cells were immunomagnetically isolated, transferred (i.p.) to new B cell-deficient mice and monitored for survival.

### Immunofluorescence Imaging

Mice brains were isolated, fixed in 4% paraformaldehyde (PFA) overnight at 4°C, and cryoprotected in a graded series of sucrose treatment (10%, 20%, and 30%, each overnight at 4°C). Para-median sagital sections were cut at 25 µm with a cryostat. Tissue sections were PAP pen applied and preblocked in serum-free protein block for 30 min at ambient temperature. Sections were then reacted overnight at 4°C with antibody against WNV antigen. After three rinses in PBS, sections were reacted with secondary antibodies conjugated with RPE for 1 hr at ambient temperature. After three additional rinses in PBS, sections were then nuclear counterstained with DAPI (Invitrogen) and mounted in fluorescence mounting medium. Images were acquired in independent channels with fluorescence microscope (Olympus BX51WI).
